# Impact of Physical Activity on Challenging Obesity in Pakistan: A Knowledge, Attitude, and Practice (KAP) Study

**DOI:** 10.3390/ijerph17217802

**Published:** 2020-10-25

**Authors:** Rizwan Ahmed Laar, Shusheng Shi, Muhammad Azeem Ashraf, Muhammad Naeem Khan, Jannat Bibi, Yibing Liu

**Affiliations:** 1 School of Sports Science and Physical Education, Nanjing Normal University, Nanjing 210023, China; rizwan_laar@yahoo.com (R.A.L.); lybnnu@163.com (Y.L.); 2 Research Institute of Educational Science, Hunan University, Changsha 410082, China; azeem20037@gmail.com; 3 Department of Sociology, School of social and Behavioral Science, Nanjing University, Nanjing 210023, China; naeem@smail.nju.edu.cn; 4 School of Physical Education, Shaanxi Normal University, Xi’an 710119, China; jannatbibi95@yandex.com

**Keywords:** KAP, Pakistan, sedentary work, PA, NCD, obesity, Yin–Yang, BMI

## Abstract

Physical activity (PA) refers to any action produced by skeletal muscle that consumes energy. According to the World Health Organization (WHO), PA is the primary element that can improve health at the community level. Obviously, PA plays an important role in the social, physical, and mental development of men and women, as well as in balancing weight. However, the large-scale negative impacts of physical inactivity on health-related issues are also recognized globally, such as obesity, which is the source of many non-communication diseases (NCDs). In Pakistan alone, 46% of deaths occur due to NCD. The majority of NCD deaths are linked to obesity, and Pakistan is the ninth most obese country in the world. Research on obesity caused by sedentary work in Pakistan is lacking, especially among university employees. To fill this gap, the current study mainly focuses on the rising non-communicable disease (NCD) rates among university employees in Pakistan due to a lack of exercise (obesity, in this case), with the help of a self-designed knowledge, attitude, and practice (KAP) questionnaire. Five universities in the Sindh province of Pakistan were surveyed (*n* = 276), following the concept of Yin–Yang as a theoretical lens. The results of the current study show that the knowledge, behaviors, and attitudes of university employees have a great influence on their body mass index (BMI). The study shows that Pakistani residents’ (especially teaching staff) perceptions and attitudes towards obesity and PA have been instructive, but their practices need to be improved.

## 1. Introduction

Physical activity (PA) refers to any physical activity produced by the skeletal muscles that consumes energy. According to the World Health Organization (WHO), the first sign of health at the community level is PA [1]. It is clear that PA plays a crucial role in social and physical development. In addition, PA has been used to maintain weight loss and weight gain and provides physical, social, emotional, and cognitive benefits [2,3]. On the other hand, governments have also recognized the large-scale negative impacts of physical inactivity on health-related problems worldwide. In developing and developed countries, chronic health conditions and non-communication diseases, such as obesity, are mainly associated with physical inactivity [4,5]. Hypertension, hyperglycemia, smoking, obesity, and a lack of physical exercise are the five major causes of death [6]. It is noteworthy that men self-reported a decrease in occupational, domestic, transportation, and leisure PA from 350 MET (metabolic equivalent) hours per week in 1997 to 253 MET hours per week in 2006. Women’s PA decreased from 390 MET working hours per week in 1996 to 246 MET working hours in 2006.

Obesity is considered to be the primary cause for many non-communication diseases (NCDs), including diabetes, hypertension, osteoporosis, and stroke [7]. Obesity is a complex disease involving excess body fat, which has a lack of physical exercise as one of the main causes [8]. As mentioned earlier, this is a medical problem that increases the risk of developing other diseases and health problems, such as heart disease, diabetes, hypertension, and some cancers [7]. Obesity is calculated by body mass index (BMI). WHO has developed different BMI guidelines for the global population and South Asia [9]. For the global population, WHO uses a number of 25 for normal BMI, which ranges from 25 to 30 for overweight, 30 to 35 for class I obesity, 35 to 40 for class II obesity, and 40 or more for obese people in class III. However, in South Asian countries, a normal BMI is 23, overweight is 23–27.5, class I obesity is 27.5–32.5, class II obesity is 32.5–37.5, and the BMI of the class III obese population is 37.5 or above (Table 1). However, the BMI does not measure body fat directly, so some people, like body-builders, may be categorized as obese even if they do not have excess body fat.

Taking into account the WHO guidelines on BMI in South Asia as a standard, the Hayatabad Medical Complex, Pakistan Endocrine Society (PES), University of Manchester, University of Glasgow, and Khyber Medical College University conducted a real-time study in Pakistan of 19,000 subjects across the country, showing that 29% of the Pakistani population is obese, with 31% suffering from class I obesity, 13% from class II obesity, and 7% from class III obesity [9]. This indicates that the obesity rate is 20% higher than that of 31% for the rest of the world (20% in class I, 7% in class II, and 7% in class III). Pakistan is the ninth most obese country in the world, and around 3.4 million Pakistani people died because of obesity in 2010 alone [10]. The risk factors of obesity have been interpreted as a high-fat diet, sedentary work, and motor transport [11]. According to Farooq et al., the main reason for the increase in obesity in Pakistan is a lack of exercise [12]. Among Pakistan’s growing population, there is an urgent need to increase the ratio of PA to reduce obesity. As Professor Dr. AH Amir from Hayatabad Medical complex told the participants at the PES’ fifth annual mid-summer endocrine conference, “This real-time research survey provides the most reliable data on obesity prevalence in Pakistan, so it should be used as a call for action” [9].

The main object of this study is to highlight the rising obesity rates among university employees in Pakistan due to a sedentary lifestyle. Then, the focus is shifted to overcoming this problem mainly by participating in physical activities. In the current study, the impact of physical activities on the obesity of university employees is measured with the help of a self-designed knowledge attitude and practice (KAP) questionnaire, which is used to enhance the knowledge, attitudes, and practices of specific themes [13,14,15,16]. We used Yin–Yang theory (discussed in the “Methods” section in more detail) as the theoretical framework of the current study, which helped in developing the questionnaire survey. The current study offers a fresh understanding of the relationship between the obesity ratio and physical activity participation rate of Pakistani university employees.

## 2. Studies on Obesity in Pakistan

Pakistan is the sixth most populous country in the world, with an urban population of 39.1% [10]. Pakistan is a developing country and is facing many crises in the field of health due to a lack of awareness. In 2015, there were around 56.4 million deaths worldwide, among which 70% died of non-communicable diseases. In total, 1.6 million died of obesity-related health problems [10]. Obesity is often accompanied by other diseases, and many studies have shown that obesity is a major cause of non-communicable diseases, leading to serious cardiovascular problems, diabetes, and premature death [17,18,19]. In Pakistan, 46% of deaths are caused by NCDs, comprising around 380,000 males and 300,000 females, and the majority of NCD deaths are related to obesity [17,18,19,20]. The main causes of overweight and obesity in Pakistan are unawareness, high-density diet consumption, and physical inactivity. Notably, studies have shown that people living in Pakistan’s big cities are more likely to be obese than those in rural areas [21]. However, there are few studies in Pakistan on the link between a lack of physical exercise and obesity, especially among university employees. A few empirical studies have been conducted [10,17], but the existing literature may oversimplify the relationship between overweight and sedentary work among university employees. Some studies have focused on the difference between overweight and obesity between urban and rural residents, finding that residents living in urban areas are more likely to be overweight or obese than residents living in rural areas, and a parallel gap was drawn between the workload of employees and sports participation [19,22].

## 3. Methods

A knowledge attitude practice (KAP) survey was conducted as the main method of the current study. The KAP survey is a quantitative method that provides access to quantitative and qualitative information. It reveals what was said, but there may be considerable gaps between what is said and what is done [23]. The KAP survey also helps to identify what is known and done about various health-related subjects and helps to measure the effectiveness of health education activities to change health-related behaviors [13,14,15,16].

### 3.1. Questionnaire Survey

A self-designed KAP questionnaire was used as a survey tool and was designed from the existing literature, previous data, and our own previous research-related experience and knowledge [13,14,15,16,24]. This questionnaire was designed in the English language, which is the second most spoken, as well as the official, language in Pakistan. We completely followed the relevant ethical considerations. All participants gave their consent for participation before the starting the survey. The protocol of the study was approved by the Institutional Review Board of School of Sports Science and Physical Education, Nanjing Normal University, China.

**Figure 1:**
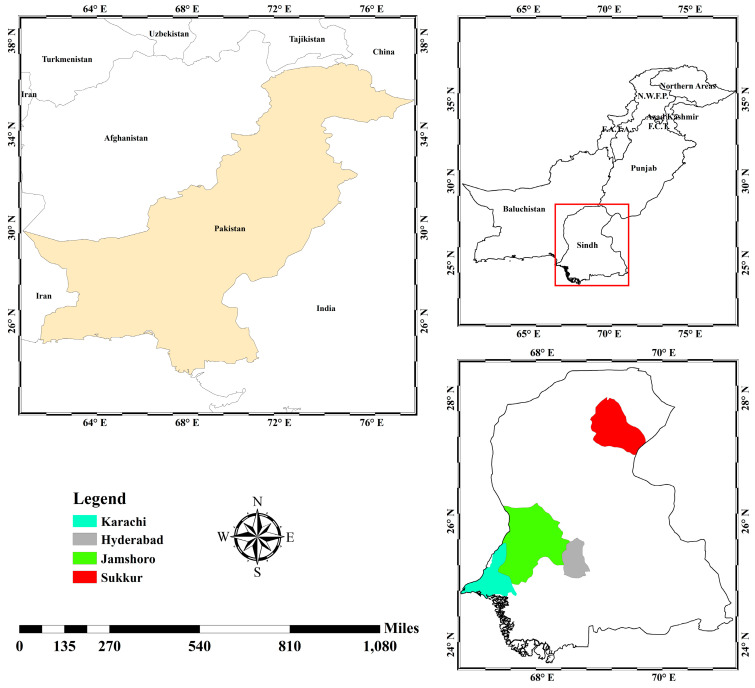
Study area map (authors’ illustration designed with ArcGIS 10.5).

During the questionnaire survey, the purpose, theme, scope, and objectives of the study were described, and it was made clear that participation was voluntary. In this study, research data were kept private, and the identities of the participants were not revealed. Each questionnaire took 15–20 min to complete for one participant. The questionnaire contained closed- and open-ended questions, such as “what are the health problems that can occur when a person is overweight or obese?”, “do you think Physical inactivity is one of the main reasons for obesity?”, “how often do you consume fast/junk/sugary/oily food or soft drinks?”, “at what time do you usually finish your dinner?”, “is PA is important to you?”, “do you participate in any PA”, and so on. Five universities (i.e., 1. Hamdard University, 2. University of Karachi, 3. Isra University, 4. University of Sindh, and 5. Sukkur Institute of Business Administration) were selected via the random sampling method. These universities are located in the four largest districts in Sindh province, which is the 2nd largest province in Pakistan (Figure 1). On this basis, scattered universities were randomly selected from the Sindh province of Pakistan. From each university, 60 employees were surveyed. First, participants were contacted through our own personal links and connections. These participants then reached out to other participants with the help of snowball sampling [24,25]. The validity measures of the current survey included training the interviewers, pilot studies, and inspecting the quality of the data collection. We measured the Cronbach’s alpha value to evaluate the reliability of the questionnaire. The value was 0.78, which indicates that the internal consistency was acceptable [26].

### 3.2. Statistical Analysis

After collecting the data and using Amos, 276 valid responses were left (*n* = 276), giving a 92% response rate (Table 2). The data were analyzed through SPSS Statistics 20 and Amos 24. Risk factor analysis and logistic regression were applied to explore the association between the obesity, BMI, and KAP scores of the participants. In the current study, we hypothesized that there would be significant differences in the level of obesity and physical activity knowledge between teaching and administration staff. We also hypothesized that people living in Pakistan’s big cities would be more likely to be obese than those in small cities and that the ratio of males in Pakistan participating in physical activities would be greater than that of females. We were inspired by the ideology of Yin–Yang, which is the theoretical lens of the current study.

### 3.3. Theoretical Framework

**Figure 2:**
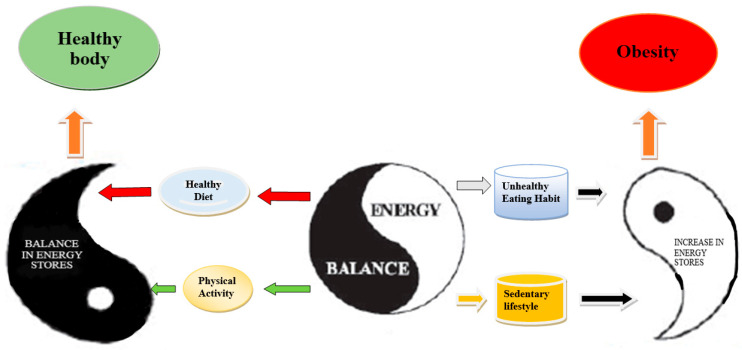
Yin–Yang model of energy balance.

Like other studies [27,28] in different countries, the current study also applies the Yin–Yang concept as a theoretical framework. Yin–Yang theory is a kind of logic that views elements in relation to their whole. Usually, Yang is associated with the functional aspect of an object and has more energetic qualities, such as moving, ascending, expanding, progressing, and acting. Yin, on the other hand, is associated with the less energetic qualities, such as stillness, descending, and under-functioning states (Figure 2). Yin and Yang also apply to the human body. In traditional Chinese medicine, good health is directly related to the balance between Yin and Yang qualities within oneself [29]. If the Yin and Yang become unbalanced, one of the qualities is considered deficient or has vacuity. Yin–Yang theory is applied to the current study through the idea that one’s eating habits and physical activity should be balanced to avoid complications such as obesity [30]. A basic scientific rule is that every action has a reaction: if a person is physically inactive and has unhealthy eating habits, their energy stores will increase, which will cause obesity, and vice versa (see Figure 2). This research provides several opportunities to understand the current situation of Pakistan, especially the participation of Pakistani university employees in physical activities and the ratio of obesity. Notably, this type of data is generally difficult to access in Pakistan, especially from female participants, which makes this material extremely valuable.

## 4. Results

### 4.1. BMI of the Sample

The BMIs of the participants were measured according to the standard values from WHO (see Table 1). More than half of the population (63%) suffers from obesity of class I, II, or III, respectively. The results also show that almost 16% of the population seems to be overweight (Table 3).

### 4.2. Association between Obesity and KAP Score

Logistic regression was applied to explore the association between obesity and KAP scores adjusted by personal characteristics. The results showed that the KAP score has significant effects on increasing or decreasing obesity among university employees. We observed that the lower scores of the female participants impacted their obesity more compared to the male participants. As the education level rises, the KAP score also rises, which results in a normal body weight. This is the reason why, in our study, the teaching staff seemed to have healthier bodies compared to the administration staff. All the results were significant (*p* < 0.05, Table 4).

### 4.3. Risk Factor Analysis

We compared the scores of the knowledge, attitudes, and practices (KAP) questions with BMI levels. A correlation matrix was used for the risk perception analysis [31,32]. In the current research, the correlation coefficient was analyzed to identify any possible significant relationships between the variables (see Table 5).

According to the results, there is a significant correlation between dietary and PA practice scores and BMI levels. This indicates that practices like not eating/eating less fast/junk/sugary/oily food or soft drinks in one’s daily diet, finishing eating dinner 6 h before sleeping, and frequently engaging in physical activities resulted in healthy individuals with a good BMI level. A significant correlation was found between the cause and prevention of obesity knowledge and the attitude scores of the participants with BMI levels. Better self-awareness regarding the risks of overweight and obesity, knowledge of the causes of overweight and obesity, the importance of PA in daily life, and maintaining a balanced diet could help to make these people healthier and stronger.

#### 4.3.1. Obesity and Physical-Activity-Related Knowledge

Table 6 shows the correct responses of the participants from knowledge section (see also [33]). The majority of university employees have a good understanding about prevention steps (65%) to address obesity and its health problems (47%). However, results show that the participants have little knowledge about physical inactivity as a reason behind obesity (Table 6).

On the other hand, to compare the knowledge levels of the teaching and administration staff, this study analyzed the correct responses of the participants related to obesity and physical activity knowledge in the shape of the mean, standard deviation, and percentage within the element. The results show that—as hypothesized—there is significant difference in the level of obesity and physical activity knowledge between teaching and administration staff (*p* < 0.05, see Table 7). The obesity and physical activity knowledge levels of teaching staff are better than those of the administration staff. This shows that the teaching staff understand the health problems of overweight and obesity and the prevention steps to overweight and obesity and consider physical inactivity to be the main reason for obesity. This is the reason why, according to the results, the teaching staff are comparatively less overweight or obese, more active in participating in physical activities, and lead healthier lives.

#### 4.3.2. Obesity and Physical-Activity-Related Attitudes

Table 8 elaborates the questions regarding participants’ attitudes towards the impact of physical activities and healthy eating habits on obesity [34]. Almost 58.70% of the population agree or strongly agree on the idea that obesity is very serious condition. Meanwhile, more than 55% of the university employees showed negative attitudes towards eating less and engaging in some physical activity or exercise (see Table 8).

Meanwhile, the mean, standard deviation, and *p* value of each variable (obesity and PA-related attitudes) were analyzed to compare the obesity and PA-related attitudes of the respondents from different cities. With the help of an independent-sample *t*-test (comparing all cities one by one with each other), we determined the significance (two-tailed) of each city. There was not a significant difference in each city’s obesity and PA-related attitude responses (*p* > 0.05 see Table 9). However, Jamshoro and Sukkur are smaller cities than Karachi and Hyderabad. Nevertheless, among the compared means between the small and big cities, no significant difference was found (*p* > 0.05) between these cities. Thus, we reject the null hypothesis (H_A_) that people living in Pakistan’s big cities are more likely to be obese than those in small cities. This result indicates that, whether they live in small or big cities, there is not a large difference between people’s ideas and attitudes towards PA and obesity (Table 9).

#### 4.3.3. Obesity and Physical-Activity-Related Practice

We have summarized the practices of university employees regarding the impact of physical activity and healthy eating habits on obesity in Table 10 (see also [34]). Results showed that most employees have fair knowledge and positive attitude about the impact of physical activity and healthy eating habits on obesity; however, they have a very low level of practicing these in their daily routines (only 15.22% participate in PA; see Table 10).

In addition, we ran crosstabs, compared the means, and performed independent-sample *t*-tests to analyze the ratio of males and females, calculate the means, and observe the significance (two-tailed), respectively. The study showed that almost 85% of the total population agrees or strongly agrees that sports is important in their lives. More than half of the population, 128 (82.5%) participants, was male, and 104 (85.9%) were female (Table 11). Despite the fact that everyone agreed or strongly agreed with the importance of PA participation, 83.9% of males and 86% of females claimed to not participate in any PA, with a *p* value greater than 0.05 (*p* > 0.05), which indicates that the groups are not significantly different. Thus, we reject our hypothesis (H_A_) that the ratio of males in Pakistan participating in physical activities would be greater than that of females. Out of the 15.2% of the population that claimed to participate in physical activities, 4.7% performed PA once a week, and only 2.2% performed it four or more times per week. Moreover, 35.5% of the male and 43% of the female population declared that fatigue from their job and their workload were the main reasons that they did not participate in physical activities. Laziness was also one of the main reasons that participants did not participate in physical activities (male = 41.3% and females = 36.6%, see Table 11). Regarding practices to control obesity with the help of diet, more than 64% of the participants ate fast/junk/sugary/oily food or soft drinks either daily or once–twice a week, comprising 68.6% of the female population and 61.9 of the male population. On the other hand, only 7.2% of the participants consumed no fast/junk/sugary/oily food or soft drinks in their diets, and only 2.2% ate their dinner between 5 and 6 p.m.

## 5. Discussion

At present, data on the knowledge, attitudes, and practices related to physical activities and obesity are still lacking. This study is the first to highlight university employees’ KAP about PA and obesity in Pakistan using the concept of the Yin–Yang model. The purpose of the current study was to identify the impact of physical activities on obesity through a KAP survey among university employees in Pakistan. According to the basic principles of the KAP survey, an improvement in knowledge will lead to a modification of attitudes and behaviors, thereby reducing the burden of disease and promoting a healthy lifestyle [11,35,36]. In addition, the KAP model emphasizes the beneficial effects of physical practices on health promotion, disease management, and risk reduction [37]. According to the WHO, the head of the household plays a major role in determining health policies for the family and society and has a significant impact on the health of current and future generations [38]. Therefore, this study selected teachers and other faculty members from different universities, who are considered as important guides that can lead their households and society towards a healthy lifestyle.

We found that the knowledge about the impact of physical activities on obesity was reasonable. This result indicates that participants understand the consequences of a sedentary lifestyle and were aware that engaging in physical activity and eating a healthy diet can overcome obesity. Only 6.8% of the respondents indicated that they did not know the correlation between physical activity and obesity. Among the respondents, the ratio of the administration staff (5.5%) was higher than that of the teaching staff (2%), possibly because the administration staff among our participants were not as highly educated or had less scientific knowledge about the human body compared to the teaching staff. According to Bookari et al. [39], knowledge can be the main factor in moving towards a healthier lifestyle. A good attitude is also the foundation of good behavior. A positive attitude helps to improve the level of knowledge and produce appropriate behavior. In this study, most of the participants showed a very positive attitude towards participating in physical activities, and most of them agreed to reduce or control their obesity levels. Positive attitude and knowledge can lead towards the Yang (functional or physically active in this case) part of the Yin–Yang concept. According to the results, there is not a significant difference in obesity and PA-related attitudes between big (Karachi and Hyderabad) and small (Jamshoro and Sukkur) cities (see Table 6). This indicates that, regardless of whether they live in small or big cities, all respondents had positive attitudes towards PA and obesity-controlling behaviors. As far as practice is concerned, we found that most of the participants (84.8%), despite their high level of knowledge and positive attitudes, did not participate in physical activities at all; the ratio of females was higher than that of males. These results contradict those of some other studies, which argue that an improvement in knowledge will lead to changes in attitudes and behaviors and that knowledge may be the main factor leading to changes in people’s behaviors and habits [11,35,36,39]. On the other hand, fewer members of the population engage in good/healthy eating habits to control overweight or obesity. For example, 64% of the participants consumed fast/junk/sugary/oily food or soft drinks either daily or once–twice a week, and most of the employees (81.5%) finished their dinner at 8–9 or after 9 p.m., which is not the healthiest time according to WHO guidelines [38]. These results support the Yin concept, which is related to less energetic qualities, sedentary lifestyle, and unhealthy eating habits. Furthermore, the results showed that only 21% of the participating university employees had a normal weight, while most of the participants (63.1%) were categorized under various classes of obesity (Class I-III). Notably, these results agree with those of some other studies [9,10], showing that 73% of the population suffers from obesity of at least one class (class I–III). This makes Pakistan the ninth most obese country in the world. The present study reveals that most university employees in the surveyed areas of Pakistan suffer from obesity problems because of their eating habits and sedentary lifestyles.

This KAP study provides a better understanding of university employees’ behaviors towards obesity and PA by implementing the concept of Yin–Yang and offers valuable information about the knowledge levels among teaching and administration staff, the attitudes of people in big and small cities, and gender-wise differences in their practical habits. These results could be used to plan lifestyle-counselling programs for employees with obesity and/or sedentary lifestyle issues. The results of this study may be significant for future obesity control and enhancing physical activity policies. Research that focuses on institutions such as universities may play a significant role in health promotion and the prevention of health problems. There are, however, some limitations to this study. This study used a KAP survey, which is a first-generation approach in health behavior research. Recently, third-generation theory-based methods and fourth-generation multi-theory-based methods have been used in health-related research. This KAP survey used predefined questions to capture information on the important knowledge, attitudes, and practices related to the most common eating habits and sedentary lifestyle issues [40]. The area (Sindh province, Pakistan) is large, but the sample size was relatively small, which could have affected the accuracy and reliability of the survey. As a result, the investigators were trained to control the data. Female participants also spoke to a male surveyor. Indeed, the female respondents, who were voluntary participants, felt relaxed and did not seem conservative when talking to the male researchers. Generally, in Pakistani society, it is difficult to obtain information from universities, especially when the participants include females. Our first attempt was met with many rejections. Lastly, the socio-cultural and religious characteristics of the selected sample were homogeneous, which may have resulted in bias. The investigators attempted to recognize any biases to avoid such results.

## 6. Conclusions

This study on university employees in Pakistan living in Sindh province is the first to use the KAP model based on Yin–Yang theory. In this study, we examined the influence of physical activity participation knowledge, attitudes, and practices (KAP) among university employees on obesity in Pakistan. The research results clearly show that university employees in Pakistan, especially teaching staff, have a good level of knowledge regarding obesity, its causes, and the consequences of physical inactivity. Most employees have a positive attitude about not being overweight or obese by maintaining healthy eating habits and know that they should participate in physical activities; however, they have a very low level of practically applying this knowledge. In terms of the Yin–Yang concept, the knowledge and attitude of Pakistani university employees towards the logic of Yin is very much clear and positive but the practice towards its functional part (Yang) needs to be improved. Furthermore, this study shows that the knowledge, practices, and attitudes of university employees largely affect their BMI status. This study shows that the awareness and attitude of Pakistani residents about obesity and PA is constantly increasing, but their practices need to be improved. Further studies should be conducted to investigate the reasons for not practicing these activities. For example, despite having positive attitudes and sufficient knowledge that participating in physical activities is good for mental and physical development and that obesity is one of the most fatal NCDs, why do Pakistani individuals not adopt healthy eating styles and participate in physical activities?

## Figures and Tables

**Table 1:** Body mass index (BMI) to diagnose obesity (global and South Asia).

BMI		Weight Status
Global	South Asia	
25	23	Normal
25–30	23–27.5	Overweight
30–35	27.5–32.5	Obesity Class I
35–40	32.5–37.5	Obesity Class II
40 or above	37.5 or above	Obesity Class III

**Table 2:** Characteristics of the sample.

Element	Frequency	Percent
Sample distribution		
Total Universities	5	100
Public	3	60
Private	2	40
Total Employees	300	100
Valid Responses (after collection and using Amos)	276	92
Age		
20–25 years	46	16.7
26–30 years	79	28.6
31–40 years	81	29.3
>40 years	70	25.4
Education		
Intermediate	68	24.6
Graduate	101	36.6
Masters	86	31.2
PhD	21	7.6
Job Position		
Teaching	87	31.5
Administration	189	68.5
Gender		
Male	155	56.2
Female	121	43.8
Marital Status		
Unmarried	188	68.1
Married	88	31.9

**Table 3:** Body mass index (BMIs) of the participants.

BMI	Frequency	Percentage
Normal	58	21.0
Overweight	44	15.9
Obesity Class I	67	24.3
Obesity Class II	64	23.2
Obesity Class III	43	15.6
Total	276	100

**Table 4:** Affiliation of the KAP score with the obesity of university employees.

Score	b	Wald_X_2	*p*	OR (95%CI)
Knowledge	0.425	9.92	0.000 ***	1.654 (1.569, 1.368)
Attitude	0.631	10.664	0.043 *	1.858 (1.511, 1.811)
Practice	0.433	5.445	0.032 *	1.870 (1.429, 1.931)

Note: Logistic regression was applied; * *p* < 0.05, *** *p* < 0.001.

**Table 5:** Correlation between the knowledge, attitude, and practice scores of the participants and their BMI levels.

Variable	Mean	±SD	Correlation	Sig.
^1^ BMI level	1.34	0.596		
Knowledge score	0.50	0.190	0.145 *	0.040
Attitude score	0.45	0.758	0.165 *	0.030
Practice score	0.65	0.430	0.138 **	0.006

Abbreviations: SD: standard deviation; Sig.: significant. * Correlation significant at a 0.05 level; ** Correlation significant at a 0.01 level. ^1^ BMI level (normal, overweight, obesity class I, obesity class II, and obesity class III; see also Table 3).

**Table 6:** University employees’ knowledge of effects of physical activities and healthy eating habits on obesity.

Category	Variable	Correct Responses	
		*n*	%
Health problems due to overweight or obesity	Increased risk of chronic conditions (such as heart/cardiovascular disease, high blood pressure and diabetes, stroke, certain types of cancer, respiratory difficulties, chronic musculoskeletal problems, skin problems, and infertility)	95	34.42
	Reduced quality of life	139	50.36
	Premature death	124	44.93
Reasons of obesity	Increased/excessive intake of energy-dense foods that are high in fat and/or sugar	130	47.10
	Decreased or lack of physical exercise	127	46.01
Preventions	Reduce energy intake (less high-energy foods and drinks)/reduce the intake of fatty and sugary foods)	182	65.94
	Eat more fruits and vegetables	173	62.68
	Eat legumes/whole-grain products more often	174	63.04
	Increase PA level/engage in regular PA	176	63.77
Physical inactivity as a reason of obesity	Yes, possibly	59	21.38
	Yes, for sure	65	23.55

Note: One score for one correct answer is given.

**Table 7:** Obesity and physical-activity-related knowledge—occupation-wise classification.

Variable	Occupation	*n*	%within ^2^ T&A	Mean	±SD	Sig.
What are the health problems that can occur when a person is overweight or obese?						
Increased risk of chronic conditions (such as heart/cardiovascular disease, high blood pressure and diabetes, stroke, certain types of cancer, respiratory difficulties, chronic musculoskeletal problems, skin problems, and infertility)	T	32	36.8	1.78	0.689	0.020
	A	63	33.3	2.02	0.831	
Reduced quality of life	T	48	55.2	1.60	0.739	0.014
	A	91	48.1	1.87	0.908	
Premature death	T	44	50.6	1.64	0.731	0.008
	A	80	42.3	1.93	0.882	
Can you tell me the reasons why people are overweight or obese?						
Increased/excessive intake of energy-dense foods that are high in fat and/or sugar	T	49	56.3	1.60	0.754	0.001
	A	81	42.9	1.96	0.907	
Decreased or lack of physical exercise	T	53	60.9	1.55	0.759	0.000
	A	74	39.2	2.00	0.887	
How can people prevent overweight and obesity?						
Reduce energy intake (less high-energy foods and drinks)/reduce the intake of fatty and sugary foods)	T	63	72.4	1.33	0.584	0.010
	A	119	63	1.59	0.825	
Eat more fruits and vegetables	T	60	69	1.37	0.593	0.011
	A	113	59.8	1.62	0.821	
Eat legumes/whole-grain products more often	T	65	74.7	1.31	0.577	0.001
	A	109	57.7	1.64	0.817	
Increase PA level/engage in regular PA	T	60	69	1.37	0.593	0.017
	A	116	61.4	1.60	0.823	
Do you think physical inactivity is one of the main reasons for obesity?						
Yes, possibly	T	34	39.1	1.61	0.491	0.000
	A	25	13.2	2.59	0.714	
Yes, for sure	T	38	43.7	1.56	0.499	0.000
	A	27	14.3	2.58	0.730	

Note: Correct responses of the participants are presented in this table (*p* < 0.05); ^2^ T = teaching staff; A = administration staff.

**Table 8:** University employees’ attitudes towards the impact of physical activities and healthy eating habits on obesity.

Variable	Strongly Disagree		Disagree		Agree		Strongly Agree	
	*n*	%	*n*	%	*n*	%	*n*	%
Are you likely to become overweight or obese?	64	23.19	61	22.10	79	28.62	72	26.09
Is it serious to be overweight or obese?	64	23.19	50	18.12	86	31.16	76	27.54
Is it good to eat less (for example, to eat a smaller portion of food)?	57	20.65	76	27.54	96	34.78	47	17.03
It is difficult for me to eat less.	54	19.57	55	19.93	83	30.07	84	30.43
It is good to do some physical activities, such as walking for 30 min a day, running, or doing sports.	47	17.03	70	25.36	91	32.97	68	24.64
It is difficult for me to do some physical activities/exercise.	37	13.41	84	30.43	91	32.97	64	23.19

**Table 9:** Multi-comparison of the obesity and physical-activity-related attitudes of respondents from big and small cities.

Variable	City								Sig.					
	Karachi		Hyderabad		Jamshoro		Sukkur							
	Mean	±SD	Mean	±SD	Mean	±SD	Mean	±SD	K&H	K&J	K&S	H&J	H&S	J&S
Are you likely to become overweight or obese?	2.53	1.069	2.67	1.123	2.69	1.226	2.46	1.078	0.432	0.410	0.702	0.956	0.321	0.317
Is it serious to be overweight or obese?	2.65	1.093	2.78	1.031	2.56	1.127	2.52	1.250	0.453	0.612	0.488	0.276	0.228	0.869
Is it good to eat less (for example, to eat a smaller portion of food)?	2.52	1.052	2.29	1.031	2.43	0.882	2.64	0.980	0.181	0.561	0.476	0.464	0.068	0.226
It is difficult for me to eat less.	2.73	1.061	2.73	1.130	2.63	1.121	2.75	1.148	0.989	0.578	0.910	0.652	0.916	0.579
It is good to do some physical activities, such as walking for 30 min a day, running, or doing sports.	2.72	1.046	2.51	1.086	2.57	1.057	2.73	0.924	0.227	0.401	0.945	0.752	0.246	0.405
It is difficult for me to do some physical activities/exercise.	2.75	0.889	2.64	1.025	2.65	1.135	2.52	0.953	0.472	0.539	0.126	0.955	0.529	0.515

Note: K = Karachi, H = Hyderabad, J = Jamshoro, and S = Sukkur (*p* > 0.05).

**Table 10:** University employees’ practice on effects of physical activities and healthy eating habits on obesity.

Variable	Positive Behavior	
	*n*	%
1. Is PA important to you?	232	84.06
2. Do you participate in any PA?	42	15.22
3. How often do you participate in sports?	42	15.22
4. How long do you participate in PA every time?	23	8.33
5. How long have you been participating in PA?	32	11.59
6. Do you think it is very hard for you to participate in physical activities?	37	13.41
7. How often do you eat fast/junk/sugary/oily food or soft drinks?	97	35.14
8. At what time usually do you finish your dinner?	51	18.48

Note: One score for one correct answer is given. Questions related to participating in PA (i.e., 3–6) were only asked of the participants who answered “yes” in question 2 (42 in this case).

**Table 11:** Obesity and physical-activity-related practices—gender-wise distribution.

Variable	Male	Female	Total	Mean ± SD
Is PA important to you?				
Strongly disagree	13	10	23	1.43 ± 0.507 *
Disagree	14	7	21	1.33 ± 0.483 *
Agree	58	52	110	1.47 ± 0.502 *
Strongly agree	70	52	122	1.43 ± 0.497 *
Do you participate in any PA?				
Yes	25	17	42	1.40 ± 0.497 *
No	130	104	234	1.44 ± 0.498 *
How often do you participate in sports?				
Once a week	6	7	13	1.54 ± 0.519 *
Once to twice a week	11	5	16	1.31 ± 0.479 *
2–3 times per week	3	4	7	1.57 ± 0.535 *
4 or more per week	5	1	6	1.17 ± 0.408 *
How long do you participate in PA every time?				
Less than 30 min	11	8	19	1.42 ± 0.507 *
30 to 60 min	10	4	14	1.29 ± 0.469 *
60 min or more	4	5	9	1.56 ± 0.527 *
How long have you been participating in PA?				
Less than three months a year	9	1	10	1.10 ± 0.316 *
Half year	5	5	10	1.50 ± 0.527 *
One year	5	6	11	1.55 ± 0.522 *
Two years	5	2	7	1.29 ± 0.488 *
More than 5 years	1	3	4	1.75 ± 0.500 *
Do you think it is very hard for you to participate in physical activities daily due to the following reasons?				
Laziness	64	37	101	1.37 ± 0.484 *
Workload at job	13	15	28	1.54 ± 0.508 *
Feeling tired after job	55	52	107	1.49 ± 0.502 *
Family pressure	23	17	40	1.43 ± 0.501 *
How often do you eat fast/junk/sugary/oily food or soft drinks?				
None	14	6	20	1.30 ± 0.470 *
Daily	39	36	75	1.48 ± 0.503 *
Once-twice a week	57	47	104	1.45 ± 0.500 *
Once-twice a month	45	32	77	1.42 ± 0.496 *
At what time usually do you finish your dinner?				
5 p.m.	2	0	2	1.00 ± 0.000 *
5–6 p.m.	4	0	4	1.00 ± 0.000 *
6–7 p.m.	25	20	45	1.44 ± 0.503 *
7–8 p.m.	70	55	125	1.44 ± 0.498 *
After 8 p.m.	54	46	100	1.46 ± 0.501 *

* Groups are not significantly different (*p* > 0.05).

## References

[1] Sadrollahi A., Hosseinian M., Alavi N.M., Khalili Z., Esalatmanesh S. (2016). Physical activity patterns in the elderly kashan population. Iran. Red Crescent Med. J..

[2] Mirsafian H., Dóczi T., Mohamadinejad A. (2014). Attitude of Iranian female university students to sport and exercise. Iran. Stud..

[3] Rasberry C.N., Lee S.M., Robin L., Laris B., Russell L.A., Coyle K.K., Nihiser A.J. (2011). The association between school-based physical activity, including physical education, and academic performance: A systematic review of the literature. Prev. Med..

[4] Blair S., Sallis R., Hutber A., Archer E. (2012). Exercise therapy–the public health message. Scand. J. Med. Sci. Sports.

[5] Paterson D.H., Jones G.R., Rice C.L. (2007). Ageing and physical activity: Evidence to develop exercise recommendations for older adults. Appl. Physiol. Nutr. Metab..

[6] World Health Organization (2010). Medical Eligibility Criteria for Contraceptive Use.

[7] (2020). Research and Education at Mayo Clinic. https://www.mayo.edu/.

[8] Alfonzo M., Guo Z., Lin L., Day K. (2014). Walking, obesity and urban design in Chinese neighborhoods. Prev. Med..

[9] Tay C. (2018). Half of Pakistan’s Population Found to Be Obese and Facing High Diabetes Risk.

[10] Siddiqui M., Hameed R., Nadeem M., Mohammad T., Simbak N., Latif A., Abubakar Y., Baig A. (2018). Obesity in Pakistan; current and future perceptions. J. Curr. Trends Biomed. Eng. Biosci..

[11] World Health Organization (2008). Putting China’s rising burden of chronic disease on the agenda. Non-Communicable Diseases.

[12] Butt F., Butt A.F., Alam F., Aslam N., Moeed H.A., Butt F.A. (2019). Perception and Management of Obesity Among Pakistani Doctors. Cureus.

[13] Alsulami A.N.J., Alshehri A.A., Alghamdi E.A., Alsefry F.S.A., Mansoor S.T. (2018). Assessment of Knowledge and Attitude and Practice of Family Towards Physical Activity in Makkah City, 2017. Egypt J. Hosp. Med..

[14] Fatema K., Hossain S., Natasha K., Chowdhury H.A., Akter J., Khan T., Ali L. (2017). Knowledge attitude and practice regarding diabetes mellitus among Nondiabetic and diabetic study participants in Bangladesh. BMC Public Health.

[15] Gillani A.H., Amirul Islam F.M., Hayat K., Atif N., Yang C., Chang J., Qu Z., Fang Y. (2018). Knowledge, attitudes and practices regarding diabetes in the general population: A cross-sectional study from Pakistan. Int. J. Environ. Res. Public Health.

[16] Ramezankhani A., Motalebi M., Tavassoli E., Heydarabadi A.B., Barekati H., Gilasi H.R., Moosavi S.A. (2013). The Study of Knowledge, attitude and practice towards physical activity and its Related Factorsof College Students Living on Campus in Shahid Beheshti University of medical science. Arch. Adv. Biosci..

[17] Choo V. (2002). WHO reassesses appropriate body-mass index for Asian populations. Lancet.

[18] Eckel R.H. (1997). Obesity and heart disease: A statement for healthcare professionals from the Nutrition Committee, American Heart Association. Circulation.

[19] Worldometers Worldometers Info Pakistan Population. https://www.worldometers.info/demographics/pakistan-demographics/.

[20] Kuczmarski R.J., Flegal K.M., Campbell S.M., Johnson C.L. (1994). Increasing prevalence of overweight among US adults: The National Health and Nutrition Examination Surveys, 1960 to 1991. JAMA.

[21] Laar R., Zhang J., Yu T., Qi H., Ashraf M.A. (2019). Constraints to women’s participation in sports: A study of participation of Pakistani female students in physical activities. J. Int. J. Sport Policy Polit..

[22] Tanzil S., Jamali T. (2016). Obesity, an emerging epidemic in Pakistan-a review of evidence. J. Ayub Med. Coll. Abbottabad.

[23] Du Monde M. (2011). The KAP Survey Model (Knowledge, Attitudes & Practices).

[24] Laar R.A., Shi S., Ashraf M.A. (2019). Participation of Pakistani Female Students in Physical Activities: Religious, Cultural, and Socioeconomic Factors. Religions.

[25] Ashraf M.A. (2019). Exploring the Potential of Religious Literacy in Pakistani Education. Religions.

[26] Tavakol M., Dennick R. (2011). Making sense of Cronbach’s alpha. Int. J. Med. Educ..

[27] Zigman J.M., Elmquist J.K. (2003). Minireview: From anorexia to obesity—The yin and yang of body weight control. Endocrinology.

[28] Yoon M.-O., Kim W.-K., Sim S.-A. (2013). Effect of the Yin-Yang Constitution Diet on Metabolic Syndrome Biomarkers in Obese Adults. J. East Asian Soc. Diet. Life.

[29] Li C.-L. (1974). A brief outline of Chinese medical history with particular reference to acupuncture. Perspect. Biol. Med..

[30] Spiegelman B.M., Flier J.S. (2001). Obesity and the regulation of energy balance. Cell.

[31] Dholakia U.M. (1997). An investigation of the relationship between perceived risk and product involvement. ACR North Am. Adv..

[32] Weerasekara P.C., Withanachchi C.R., Ginigaddara G., Ploeger A. (2020). Food and Nutrition-Related Knowledge, Attitudes, and Practices among Reproductive-Age Women in Marginalized Areas in Sri Lanka. Int. J. Environ. Res. Public Health.

[33] Zeng Y., Hu X., Li Y., Zhen X., Gu Y., Sun X., Dong H. (2019). The quality of caregivers for the elderly in long-term care institutions in Zhejiang Province, China. Int. J. Environ. Res. Public Health.

[34] Tang Q., Lin Q., Yang Q., Sun M., Liu H., Yang L. (2020). Knowledge, Attitude, and Practice of Adolescent Parents on Free Sugar and Influencing Factors about Recognition. Int. J. Environ. Res. Public Health.

[35] Kigaru D.M.D., Loechl C., Moleah T., Macharia-Mutie C., Ndungu Z.W. (2015). Nutrition knowledge, attitude and practices among urban primary school children in Nairobi City, Kenya: A KAP study. BMC Nutr..

[36] Xu X., Chen C., Abdullah A.S., Liu L., Sharma M., Li Y., Zhao Y. (2016). Smoking related attitudes, motives, and behaviors of male secondary school students in an urban setting of China. SpringerPlus.

[37] Kamp B. (2010). Position of the American Dietetic Association, American Society for Nutrition, and Society for Nutrition Education: Food and nutrition programs for community-residing older adults. J. Am. Diet. Assoc..

[38] World Health Organization (2000). Healthy Nutrition: The Role of Women: Report on a WHO Meeting, Murmansk, Russian Federation 14–15 June 2000.

[39] Bookari K., Yeatman H., Williamson M. (2016). Exploring Australian women’s level of nutrition knowledge during pregnancy: A cross-sectional study. Int. J. Womens Health.

[40] Mohd Shariff Z., Lin K.G., Sariman S., Lee H.S., Siew C.Y., Mohd Yusof B.N., Mun C.Y., Mohamad M. (2015). The relationship between household income and dietary intakes of 1–10 year old urban Malaysian. Nutr. Res. Pract..

